# Complete genome sequence of *Bacillus pumilus* LBUM494, a plant-beneficial strain isolated from the rhizosphere of a strawberry plant

**DOI:** 10.1128/mra.00825-24

**Published:** 2024-09-09

**Authors:** Adrien Biessy, Martin Filion

**Affiliations:** 1 Saint-Jean-sur-Richelieu Research and Development Centre, Agriculture and Agri-Food Canada, Saint-Jean-sur-Richelieu, Quebec, Canada; 2 Department of Plant Science, McGill University, Macdonald Campus, Ste. Anne de Bellevue, Quebec, Canada; Department of Biology, Queens College, Queens, New York, USA

**Keywords:** *Bacillus*, plant-beneficial

## Abstract

Here, we present the complete genome sequence of *Bacillus pumilus* LBUM494, a plant-beneficial bacterial strain isolated from the rhizosphere of a strawberry plant grown in an agricultural field located in Bouctouche, New Brunswick, Canada. The genome size is 3,699,913 bp with a GC content of 41.7%.

## ANNOUNCEMENT

Numerous *Bacillus pumilus* strains have been shown to promote plant growth using several mechanisms (1), notably through the production of bioactive gibberellins (2, 3). Expanding the genomic resources of this species will contribute to a better understanding of the plant growth promotion mechanisms at play and support the development of tailored microbial inoculants. Here, we report the complete genome sequence of *B. pumilus* LBUM494. This strain was isolated in 2005 from the rhizosphere of a strawberry plant (*Fragaria* × *ananassa* Duch) grown in an agricultural field located in Bouctouche, New Brunswick, Canada (46.432232–64.769877). Rhizosphere soil samples were collected by harvesting the soil adhering to the root system. Samples were kept at 4°C. One gram of soil was suspended in 100 mL of a 0.9% NaCl solution and agitated for 5 min at 250 rpm. The suspension was serially diluted and plated onto tryptic soy agar (BD Difco). The plates were incubated at 25°C for 48 h, and isolated colonies were subsequently purified on the same medium. Bacteria were kept at −80°C in 25% glycerol.

LBUM494 was grown in tryptic soy broth (BD Difco) for 24 h at 25°C, and genomic DNA was extracted using the DNeasy UltraClean Microbial kit (Qiagen) following the manufacturer’s instructions. Library preparation and genome sequencing were performed at the Integrated Microbiome Resource (Halifax, NS, Canada). Genomic DNA was mechanically sheared and size-selected using g-TUBE devices (Covaris). The resulting DNA fragments (~8 to 10 kb) were cleaned up and converted into a WGS library using the SMRTbell Prep Kit v.3.0 (Pacific Biosciences) following the manufacturer’s instructions. Genome sequencing was performed on a Sequel II sequencer (Pacific Biosciences) using a SMRT Cell 8M with v.2.0 chemistry, generating 39,711 HiFi reads with a read *N*_50_ value of 7,935 bp (242,921,274 nucleotides in total). The quality of the raw reads was checked using FastQC v.0.11.9 (4). The genome of LBUM494 was assembled into a single chromosome with Flye v.2.9-b1768 (5) using the command “--pacbio-hifi.” The mean read coverage was 64-fold. The chromosome was automatically circularized by the Flye algorithm and rotated using Geneious Prime v.2024.0.7 (Biomatters). The origin of the sequence was set at the start of the *dnaA* gene. The genome was annotated by the National Center for Biotechnology Information Prokaryotic Genome Annotation Pipeline v.6.7 (6). Defaults parameters were used for all software unless otherwise specified. Sequencing and assembly metrics, as well as genome features, are presented in Table 1.

**TABLE 1:** Genome sequencing/assembly metrics and genome features

Metric	Data for strain LBUM494
No. of reads	39,711
Read *N*_50_ (bp)	7,935
Coverage (*x*)	64
Genome size (Mb)	3,699,913
GC content (%)	41.7
No. of CDSs^a^	3,655
No. of pseudogenes	33
No. of rRNAs	24
No. of tRNAs	81
GenBank accession no.	CP162095
Sequence Read Archive accession no.	SRR29881130

^a^ CDS, coding DNA sequence.

**Fig 1:**
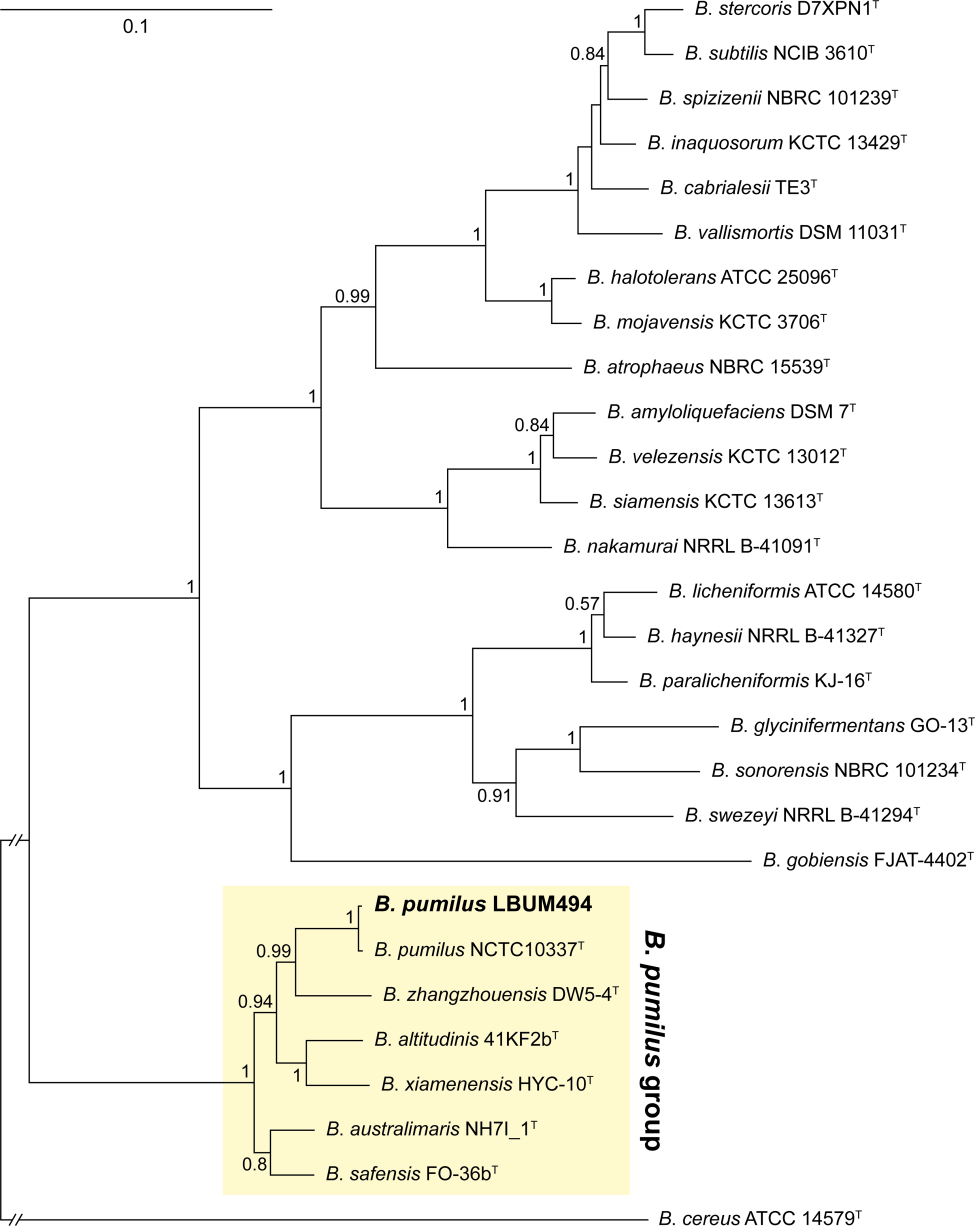
Phylogenetic placement of LBUM494 within the *B. subtilis* species complex. The genomes of *Bacillus*-type strains belonging to the *B. subtilis* species complex (and the outgroup *Bacillus cereus* American Type Culture Collection 14579^T^) were downloaded from GenBank. The complete nucleotide sequences of four housekeeping genes (*gyrA*, *gyrB*, *rpoB*, and *rpoC*) were extracted from each genome, concatenated, and subsequently aligned using Clustal Omega v.1.2.2 (8). The resulting alignment was used to generate a phylogenetic tree using FastTree v.2.1 (9) and the Juke-Cantor model. The tree topology was verified by computing Shimodaira Hasegawa support values. Only support values above 0.5 are displayed at the nodes. The scale bar indicates sequence divergence. The strain under study is highlighted in bold.

The genome size of LBUM494 is 3,699,913 bp, with a GC content of 41.7% (Table 1). The genome contains 3,655 coding DNA sequences, 33 pseudogenes, 24 rRNA, and 81 tRNAs. To visualize the phylogenetic placement of LBUM494 within the *Bacillus subtilis* species complex, we performed a multilocus sequence analysis (Fig. 1). LBUM494 clustered with *B. pumilus* NCTC10337^T^ (LT906438). Species-level identification was performed using the Type (Strain) Genome Server (7) on 16 July 2024. The digital DNA-DNA hybridization (dDDH) value between LBUM494 and NCTC10337^T^ was calculated using the *d*_4_ formula. LBUM494 was found to belong to the species *B. pumilus* (dDDH = 87.2% with NCTC10337^T^).

## Data Availability

The complete genome of *Bacillus pumilus* LBUM494 (BioProject PRJNA1136810) has been deposited in GenBank under accession number CP162095. The version described in this paper is the first version. The raw sequencing data have been deposited to the Sequence Read Archive under accession number SRR29881130.
